# Co-creation of an exercise inventory to improve scapular stabilization and control among individuals with rotator cuff-related shoulder pain: a survey-based study amongst physiotherapists

**DOI:** 10.1186/s40945-022-00132-7

**Published:** 2022-04-12

**Authors:** Marc-Olivier Dubé, Jasmine Arel, Philippe Paquette, Jean-Sébastien Roy, François Desmeules, Dany H. Gagnon

**Affiliations:** 1 Center for Interdisciplinary Research in Rehabilitation and Social Integration, Quebec City, Quebec, G1M 2S8 Canada; 2 Department of Rehabilitation, Faculty of Medicine, Université Laval, Quebec City, Quebec, G1R 1P5 Canada; 3 Centre for Interdisciplinary Research in Rehabilitation of Greater Montreal, Centre intégré universitaire de santé et services sociaux du Centre-Sud-de-l’Île-de-Montréal, Institut universitaire sur la réadaptation en déficience physique de Montréal, 6300 Avenue Darlington, Montreal, Quebec, H3S 2J4 Canada; 4 School of Rehabilitation, Faculty of Medicine, Université de Montréal, Montreal, Quebec, Canada; 5 Orthopaedic Clinical Research Unit, Maisonneuve-Rosemont Hospital Research Center, Montreal, Canada

**Keywords:** Exercises, Pain, Physical therapy, Rehabilitation, Rotator cuff, Scapula, Shoulder

## Abstract

**Background:**

Scapular stabilization exercises (SSE) are often included in the treatment of individuals with rotator cuff-related shoulder pain (RCRSP) to decrease pain and improve function. These SSE typically aim to strengthen the scapular muscles and optimize dynamic neuromuscular control of the scapula, which may improve overall shoulder stability and movement quality. No consensus of the recommended SSE for the management of RCRSP is available. Hence, this study aimed to consult physiotherapists to co-create an inventory of recommended SSE based on the exercise’s relevance and frequency of prescriptions for the rehabilitation of individuals with RCRSP.

**Methods:**

A group of 16 physiotherapists with experience in treating shoulder pain participated in a sequential consultation incorporating two distinct rounds of consultation focusing on SSE (modified Delphi design). In round 1, physiotherapists identified and demonstrated up to 10 SSE that they commonly recommend or use among individuals with RCRSP. The description and performance of all SSE were audio and video recorded. All SSE suggested by more than one participant in round 1 advanced to round 2. In round 2, physiotherapists rated these SSE on a 4-point Likert scale according to their perceived relevance and frequency of prescription for this population.

**Results:**

In round 1, out of the 25 SSE recommended by participants, 19 SSE (76.0%) were recommended by more than one physiotherapist and advanced to round 2. In round 2, 13 SSE were consensually classified (agreement ≥75%) as being relevant for the rehabilitation of individuals with RCRSP. SSE targeting the recruitment of the serratus anterior and lower trapezius muscles were considered the most relevant for the management of RCRSP, whereas SSE targeting neuromuscular scapular control were the most prescribed SSE for the management of RCRSP.

**Conclusions:**

An inventory composed of 13 SSE was co-created by physiotherapists based on their relevance and frequency of prescription for the rehabilitation of individuals with RCRSP. When designing an exercise program, physiotherapists can use this SSE inventory to inform their exercise selection, in combination with their current knowledge on shoulder rehabilitation, as well as patients’ preferences.

**Supplementary Information:**

The online version contains supplementary material available at 10.1186/s40945-022-00132-7.


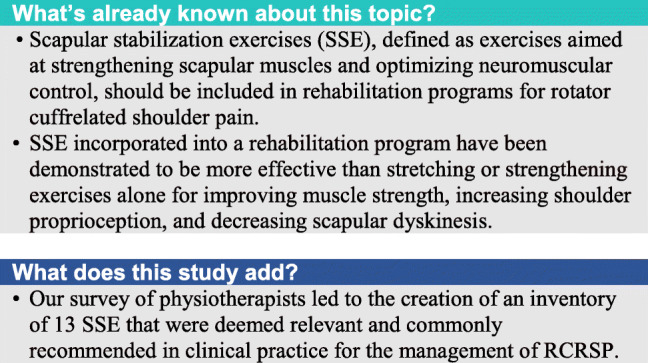


## Background

Rotator cuff-related shoulder pain (RCRSP) is the most common shoulder disorder, representing 40 to 60% of all medical consultations motivated by shoulder pain [1–3]. The pain and functional limitations reported by individuals with RCRSP may be explained in part by mobility-related deficits observed during arm movements, such as abnormal glenohumeral (GH) or scapulothoracic (ST) kinematics, as well as by performance deficits of the rotator cuff (RC) and scapular muscles [4, 5]. Conservative rehabilitation is the primary approach to address these deficits and to restore shoulder function [6]. Optimal shoulder function depends, amongst other things, on adequate interaction between active (RC and ST muscles) and passive (GH ligaments and capsule as well as bony structures) components to produce movement [7]. Weakness or dysfunction of any RC or ST muscles may generate muscle imbalance which, in turn, may compromise kinematics and alter shoulder function [1, 8–12]. Conservative rehabilitation programs, which commonly include strengthening and motor control exercises targeting RC (supraspinatus [SS], infraspinatus [IS]) and/or ST (upper trapezius [UT], middle trapezius [MT], lower trapezius [LT], serratus anterior [SA]) muscles, are known to be effective in reducing RCRSP-associated deficits and optimizing shoulder function [3, 13–18].

Several studies [19–26] have suggested that scapular stabilization exercises (SSE), defined as exercises aimed at strengthening scapular muscles and optimizing neuromuscular control, should be included in rehabilitation programs for RCRSP [24, 27]. In fact, SSE incorporated into a rehabilitation program have been shown to be more effective than stretching or strengthening exercises alone for improving muscle strength, increasing shoulder proprioception, and decreasing scapular dyskinesis [20, 28, 29]. Significant changes in static scapular positioning and in scapulothoracic mobility have also been reported after an SSE program [20]. While multiple SSE are currently proposed for the rehabilitation of RCRSP [19–23, 25], no consensus has been established regarding the most commonly recommended exercises for the management of RCRSP [24, 26].

Given the number of SSE inventoried in the literature, physiotherapists face challenges in deciding which SSE to recommend to individuals with RCRSP in clinical practice. To better inform physiotherapists’ clinical decision-making process, the most commonly recommended SSE for the management of RCRSP should be documented. Therefore, this study aimed to consult physiotherapists to co-create an inventory of commonly recommended SSE based on their relevance and frequency of prescription for the rehabilitation of individuals with RCRSP. It was anticipated that this study would yield to the co-creation of an inventory of at least 10 different SSE that are commonly used by physiotherapists in clinical practice for the treatment of RCRSP.

## Methods

### Study design

The present study was operationalized around a sequential two-round consultation process that solicited a panel of physiotherapists (modified Delphi design). To achieve a consensus on a relevant exercise for the management of RCRSP, each recommended SSE had to obtain a relevance score of 2 (relevant) or 3 (highly relevant) on a 4-point Likert scale (0–3) by ≥75% of the participants [30].

### Participants

Physiotherapists with experience in treating individuals with shoulder pain and expressing a special interest in shoulder rehabilitation were recruited through an initial outreach flyer sent via the mailing list of the physiotherapy program at the *Université de Montréal* (Montreal, Canada) and through the snowball sampling recruitment technique which involved asking recruited participants to help identify other potential participants. To be included in the study, physiotherapists had to have at least four years of clinical experience working with individuals with musculoskeletal disorders and dedicate at least 20% of their weekly workload to individuals experiencing RCRSP. Following an initial phone screening, where detailed information about the research protocol was presented, all participants gave their written consent prior to the initiation of the study. Ethical approval was obtained from the Research Ethics Board of the Centre for Interdisciplinary Research in Rehabilitation of Greater Montreal (CRIR-1187-1116).

### Sample size

According to Clayton et al., [31] it is recommended to recruit a group of 10 to 15 participants to reach saturation for the study design adopted. Considering an expected loss to follow-up of 15%, a sample size of 16 physiotherapists was deemed appropriate to reach the objective of this study.

### Physiotherapists consultations

#### Round 1: initial inventory of all SSE recommended by physiotherapists

Round 1 aimed to identify the SSE recommended by each participating physiotherapist for the rehabilitation of individuals with RCRSP. To do so, a member of the research team (J.A.) met the participating physiotherapists face to face individually at their clinical setting. During a 60-min meeting, a clinical vignette presenting a typical case of RCRSP was provided and read to each participant to stimulate their reflection on the SSE they often prescribe. To ensure clarity and adequate clinical representation of individuals with RCRSP, the clinical vignette was independently reviewed by two experienced physiotherapists in shoulder rehabilitation before the initiation of the study. These reviewers were not members of the research team or the panel of participants. After the clinical vignette was presented, an individual semi-structured interview took place with each participant, during which the participant described and demonstrated five to 10 SSE commonly recommended to individuals with RCRSP. For each of these SSE, participants were also invited to comment further on and demonstrate their progression, whenever applicable. All descriptions and demonstrations were audio and video recorded.

All SSE described and demonstrated by all participants were analyzed and computed by research team members (J.A., P.P., and D.H.G.). When differences among the SSE were observed, the following parameters were used to highlight similarities among them: a) individual position; b) upper limb position and movement; c) instructions. Once similarities were confirmed across SSE, they were considered as a variant from one another and analyzed as a single SSE. All SSE that were proposed by at least two participants were automatically “preselected” and progressed to round 2. Before doing so, all preselected SSE were audio and video-recorded by the research team, using an independent person as a model and another one as a narrator, to provide detailed instructions and information about each SSE and their progressions.

#### Round 2. Classifying the most commonly recommended SSE

Round 2 aimed to evaluate the relevance and frequency of usage of the most commonly recommended SSE from round 1. For each SSE that progressed to round 2, each participant was invited to watch a video that presented the SSE and its potential progression before answering an online questionnaire. The online questionnaire aimed to assess their perceived relevance of the proposed SSE for the rehabilitation of individuals with RCRSP and anticipated frequency at which the SSE would be recommended or used in clinical practice. The perceived relevance was evaluated using a 4-point Likert scale (0, non-relevant; 1, somewhat relevant; 2, relevant; 3, highly relevant). The perceived frequency of prescription was evaluated using another 4-point Likert scale (0, never; 1, rarely; 2, often; 3, always). Participants were given a 10-day period to watch the videos and answer the online questionnaires.

### Statistical analysis

Descriptive statistics were used to summarize demographic characteristics of participants. Mean and standard deviation were calculated from the 4-point Likert scales results in round 2. A total score for each SSE was calculated using the sum of scores and analyzed regarding the relevance and frequency of prescription. Only the SSE that were scored ≥2 (relevant or highly relevant) by 75% (12/16 participants) of the panel for relevance were included in the final SSE inventory. This threshold indicated that a consensus had been reached among participants. All data analyses were performed using SPSS Statistics 20 for *Windows* (IBM Corp., Armonk, USA).

## Results

### Participants

A total of 16 physiotherapists completed both rounds (100% retention rate) (Table 1). As entry-to-practice standards, most physiotherapists (*n* = 10, 62.5%) had completed a bachelor in physiotherapy (graduated before 2011), whereas the others (*n* = 6, 37.5%) had completed a clinical master’s degree in physiotherapy (graduated 2011 and after). On average, participants had gained 11.7 ± 8.6 years of clinical experience (range: 4–33 years) and individuals with shoulder pain seeking rehabilitation represented 28.1 ± 12.8% (range: 20–70%) of their weekly caseload.

**Table 1:** Participant’s demographic

	Participants (***N*** = 16)
Mean Age ± SD (years)	36.1 ± 8.8
Gender (Female/Male)	11/5
Professional experience (years)	
4–9	7
10–14	5
15+	4
Percentage of clients with shoulder pain in weekly caseload	
20–29	10
30–39	4
40+	2

### Co-creation process

During round 1, participants presented an average of six different SSE. A total of 25 SSE (including variations of the same exercise) were suggested and demonstrated by the participants, among which 19 SSE were presented by two or more participants and advanced to round 2. Amongst those 19 SSE, slight variations and progressions existed, such as prone vs. sitting position or resistance type. As previously described, when exercises were deemed to be variations of the same base exercise, they were pooled together and counted as a single SSE. All exercises included in round 1 are presented in Supplementary File 1.

**Fig. 1:**
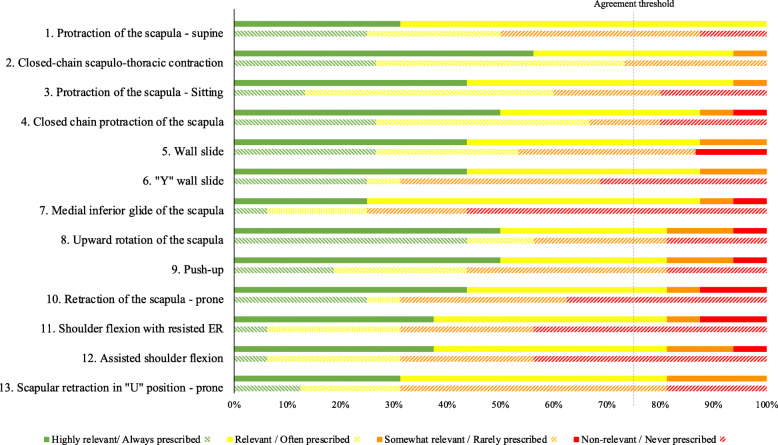
Percentage frequency distributions that specify the percentage of observations that exist for each relevance (solid colors) and frequency (dashed colors) levels defined hereunder for each SSE advanced to round 2. The 75% agreement threshold line also confirms the relevance of the SSE included in the final inventory. *ER = external rotation

During round 2, 13 out of the 19 SSE were perceived as being relevant or very relevant by more than 75% of participants, reaching the consensus threshold. Frequently prescribed SSE predominantly target activation of the SA muscle (i.e., *Exercises 1, 2, 3, 4, 5, 6 and 9 in* Fig. 1) whereas some others predominantly target the trapezius muscles (i.e., *Exercises 7,8, 10 and 13 in* Fig. 1). The complete ranking of SSE, based on the total scores and according to the results regarding relevance and frequency of prescription, is available in Fig. 1.

## Discussion

### What SSE are perceived as being the most relevant for the treatment of individuals with RCRSP?

Scapulothoracic muscles are crucial for restoring shoulder stability and optimizing scapular and glenohumeral control [32]. Exercises targeting the shoulder muscles, such as SA and LT, have often been recommended for this purpose [32]. Participant panel of physiotherapists recognized the relevance of these muscles as evidenced by the number of exercises targeting scapular protraction (3/13) or wall slide (3/13) in the inventory. Interestingly, the SSE included in the proposed inventory differ in some aspects from those proposed in recent studies [19, 21, 24, 33]. For example, Kibler et al. proposed three closed-chain scapular exercises to stimulate depression, retraction, and protraction of the scapula [24]. Whereas, in this study, the panel of participants proposed a mix of open and closed-chain exercises to stimulate those same scapular movements. In addition, Cools et al. suggested forward shoulder flexion in a side-lying position with a weight in the hand, shoulder external rotation with a weight in the hand in side-lying position, and prone shoulder extension with a weight in the hand [21]. In contrast, most of the exercises suggested by our panel of physiotherapists were performed in a standing or sitting position, which better reflects the demands of everyday tasks. Those differences with the literature could be a by-product of the clinical preferences and experience of the physiotherapists surveyed in the present study, who may put an emphasis on functional exercises incorporating forward reaching movements. This is relevant with individuals with RCRSP as they may present with different alterations in their ST kinematics [18].

### What are the most frequently prescribed exercises for this population?

Previous studies have suggested that rehabilitation exercises for RCRSP should aim to increase neuromuscular control of the shoulder by restoring balance between SA/LT muscles and UT muscle, since increased UT activation may contribute to altered scapular motion [9, 12, 21, 34–39]. This emphasis on SA muscle activation most likely translates into the *closed-chain scapulo-thoracic contraction exercise* (Exercise 4) being perceived as both relevant and frequently recommended in clinical practice by the physiotherapists consulted.

It is also important to highlight that the effectiveness of an exercise may depend on the quality of the movement performed [40]. Indeed, it is not simply because the recruitment of a muscle is targeted during specific strengthening exercises that the individual will recruit it more during functional movements. It is therefore necessary to consider both of these concepts when prescribing exercises. This may explain why exercises addressing scapular neuromuscular control, such as *closed-chain protraction of the scapula* (Exercise 4) and *wall slide* (Exercise 5) are among the most recommended SSE by physiotherapists.

Of note, many of the exercises identified as being the most relevant were not those identified as being the most frequently prescribed by the physiotherapists surveyed. One possible explanation behind this discrepancy is that although there exists a good rationale for prescribing certain exercises (e.g. aiming to bias specific muscles when performing a movement), the choice of exercises is ultimately tailored specifically for each individual. Also, some exercises could have been identified as relevant based on their rationale, but physiotherapists might prefer other therapeutic exercises or activity-based exercises which served the same purpose. Patients’ preferences may also inform the selection of exercises prescribed by the rehabilitation professionals as they are a key component of evidenced-based practice.

### In which positions should SSE be performed?

Of the exercises reported by the physiotherapy, 76.9% of the exercises were performed at ≤90° arm elevation. This is consistent with findings from Wright et al., who reported in their 2017 systematic review of exercises used in overhead athletes, that the strongest available evidence (Grades B and C) supported exercises performed at ≤90° arm elevation [41]. However, they also advocated for the use of exercises at ≥90° arm elevation since those better represent activities of daily life and sports requirements.

Exercises in side-lying position such as *forward shoulder flexion* and *shoulder external rotation* have been suggested to provide a low UT/LT ratio by optimally recruiting the MT or LT muscles with minimal activation of UT muscle [21]. In addition, *side-lying shoulder external rotation* exercise is reported to enhance activation of SS, IS, MT, and posterior deltoid (PD) muscles [42] but, when it is performed in prone position, it is believed to enhance activation of the LT muscle [43]. While exercises in a prone or side-lying position have been suggested in the literature based on muscle activity measured with electromyography (EMG), it is important to prescribe exercises that are done in functional positions, such as standing, to better reflect the demands of activities of daily life. This emphasis can be observed in the exercises that make up the SSE inventory as 69.2% were performed in a standing position. Completing several different exercises can optimize and consolidate motor learning and thus facilitate the transfer of the newly acquired spectrum of skills into activities of daily living [44].

### Clinical recommendations

This SSE inventory is meant to be used by rehabilitation professionals, particularly physiotherapists, to facilitate their selection of SSE for the management of individuals with RCRSP. Exercises targeting SA and LT muscles recruitment were considered the most relevant and recommended for the rehabilitation of RCRSP. Selection and progression of each SSE should be based on individualized parameters stemming from the clinical evaluation performed by the rehabilitation professional and the clients’ perspectives to optimize the clinical effectiveness of their rehabilitation programs. In addition, the inventory of SSE should be used as a complement to habitual therapeutic exercises selected whenever a strength or neuromuscular control deficit is identified during the evaluation. Prescribed exercises should aim to improve individuals’ functional level. For instance, it is not recommended to prescribe all SSE to the same individual, as treatment compliance is favored when fewer exercises are prescribed, rather than many [45, 46]. Conversely, clinicians should not minimize other exercises or interventions that have been shown to be effective in the management of RCRSP, such as education and exercises targeting GH and core muscles [47–49]. Finally, when prescribed to an individual as part of a home-exercises program, SSE should be demonstrated by the rehabilitation professional and should come with clear instructions and specific exercises parameters (e.g., position, tempo, repetitions, sets, load) at an adequate level.

### Strengths and limitations

To our knowledge, this is the first study presenting a physiotherapist survey on the relevance and frequency of prescription of SSE in an effort to co-create an SSE inventory for the rehabilitation of individuals with RCRSP. However, we recognize some limitations in this study. This modified Delphi was conducted only amongst physiotherapists working in the Montreal region with most of them having also been trained in the Montreal region (i.e., potential regional preference bias). Some of the physiotherapists who participated in the study were also treating individuals with shoulder pain to a limited extent in their clinical practice (i.e., potential expertise bias). Hence, it would have been interesting to recruit participants from different regions to provide broader perspectives and to recruit physiotherapists dedicating a greater proportion of their time treating individuals with shoulder pain to strengthen the level of expertise. In addition, the SSE inventory’s clinical acceptability by individuals with RCRSP was not evaluated. When developing interventions, it is important to consider patients’ preferences and beliefs, as they are an integral part of the evidence-based practice model. Future studies are needed to verify how the performance of the SSE included in this inventory affect the absolute and relative muscle demands and synergies at the shoulder (i.e., mechanistic effects) and to determine whether the performance of these SSE leads to clinically and meaningful beneficial changes among individuals with RCRSP (i.e., intervention effects).

## Conclusion

A panel of physiotherapists with experience in treating individuals with shoulder pain reached a consensus over a comprehensive list of 13 SSE deemed relevant and commonly recommended in clinical practice for the management of RCRSP. The inventory of SSE created in this study can support in part the decision-making process of rehabilitation professionals when incorporating the performance of SSE within their intervention. For the rehabilitation process to be optimal, the intervention needs to be informed by the expertise of the rehabilitation professionals (e.g., knowledge on shoulder biomechanics) and the perspectives of individuals with RCRSP seeking treatment. Future studies are needed to strengthen evidence regarding the proposed exercise inventory to improve scapular stabilization and control.

## Supplementary Information

**Additional file 1.** Supplementary File 1: SSE inventory.

## Data Availability

The datasets used and/or analyzed during the current study are available from the corresponding author upon reasonable request.

## Abbreviations

SSE: Scapular stabilization exercises
RCRSP: Rotator cuff-related shoulder pain
GH: Glenohumeral
ST: scapulothoracic
RC: Rotator cuff
SA: Serratus anterior
LT: Lower trapezius
MT: Middle trapezius
UT: Upper trapezius
SS: Supraspinatus
IS: Infraspinatus
PD: Posterior deltoid
