# Supplementary material for: Co-creation of an exercise inventory to improve scapular stabilization and control among individuals with rotator cuff-related shoulder pain: a survey-based study amongst physiotherapists
Source: Arch Physiother. 2022 Apr 12;12:11. doi: 10.1186/s40945-022-00132-7 (PMC9003989; doi:10.1186/s40945-022-00132-7)
Supplement: Supplementary file 1 — Additional file 1. Supplementary File 1: SSE inventory. [file 40945_2022_132_MOESM1_ESM.docx]

| **Supplementary File 1: SSE inventory** | | |
| --- | --- | --- |
| **Initial position and execution** | **Exercises** | **Progressions** |
| **Selected exercises for the SSE inventory** | | |
| **1. PROTRACTION OF THE SCAPULA – SUPINE** | 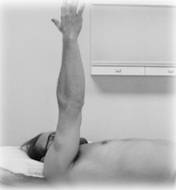 | 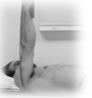 |
| **EXERCISE:**  Starting position: Patient supine with shoulder at 90° of flexion.  Execution: Protraction of the scapula. Palpation of the pectoralis minor can be done simultaneously to ensure that there is no contraction. Patient comes back to initial position and repeats.  **PROGRESSION:**  Same movement execution with an elastic band added parallel to the upper limb and held in patient’s hand. |  |  |
| **2. CLOSED-CHAIN SCAPULO-THORACIC CONTRACTION** | 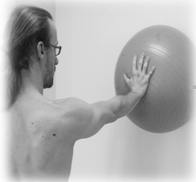 | 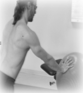 |
| **EXERCISE:**  Initial position: Patient standing facing a wall, shoulder at 90° of flexion, holding a medicine ball between his hand and the wall. Execution: Patient draws letters of the alphabet on the wall with the medicine ball. **PROGRESSION:**  Same movement execution, medicine ball on a 45° plan relative to the patient. |  |  |
| **3. PROTRACTION OF THE SCAPULA – SITTING POSITION** | 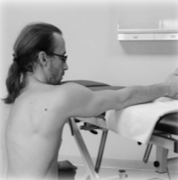 | 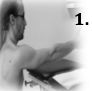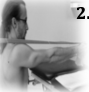 |
| **EXERCISE:**  Initial position: Patient sitting with shoulder at 90° of flexion, supported on a flat surface. Execution: Protraction of the scapula. Patient comes back to initial  position and repeats.  **PROGRESSION 1:**  Same movement execution, arm not supported.  **PROGRESSION 2:**  Same as progression 1, with an elastic band parallel to the upper limb and held in patient’s hand. |  |  |
| **4. CLOSED-CHAIN PROTRACTION OF THE SCAPULA** | 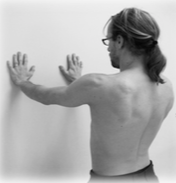 | 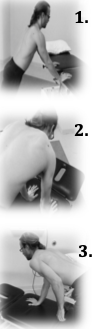 |
| **EXERCISE:**  Initial position: Patient standing facing a wall, shoulders at 90° of flexion, hands on the wall. Trunk and legs parallel to the wall. Execution: Patient pushes on the wall in order to protract the scapula and comes back to initial position or slightly retracts the scapula and repeats.  **PROGRESSION 1:**  Same movement execution, on a 45° plan relative to the patient.  **PROGRESSION 2:**  Same as progression 1, patient in four-legged position  **PROGRESSION 3:**  Same as progression 2 without stance on non-painful upper limb. |  |  |
| **5. WALL SLIDE** | 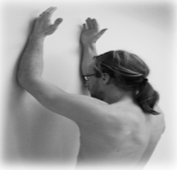 | |
| **EXERCISE:**  Initial position: Patient standing facing a wall, shoulders at 110° of flexion, ulnar side of the forearm on the wall. Trunk and legs parallel to the wall.  Execution: Patient slides their forearms above the head, in flexion, through maximal pain-free range of motion, then returns to initial position and repeats. |  |  |
| **6. “Y” WALL SLIDE** | 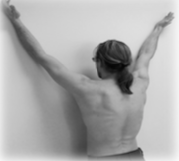 | |
| **EXERCISE:**  Initial position: Patient standing facing a wall, shoulders at 30° of flexion and external rotation, ulnar side of the forearm on the wall. Trunk and legs parallel to the wall. Execution: Patient slides their forearms above the head, in order to slide in flexion and abduction of shoulders, through maximal pain-free range of motion, then returns to initial position and repeats. |  |  |
| **7. MEDIAL INFERIOR GLIDE OF THE SCAPULA** | 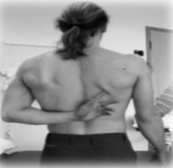 | |
| **EXERCISE:**  Initial position: Patient standing neutral. Execution: Patient medially and inferiorly glides the scapula in an isometric contraction. Feedback at the inferior border of the scapula can be done with the contralateral hand if shoulder internal rotation is sufficient. No movement is required. |  |  |
| **8. UPWARD ROTATION OF THE SCAPULA** | 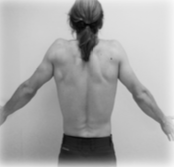 | |
| **EXERCISE:**  Initial position: Patient standing, shoulders at 30° of abduction.  Execution: Patient brings their shoulder to their ears, while arms are slightly in abduction, in order to create an upward rotation of the scapula. Patient stops shoulders motion at mid-range between shoulder depression and full elevation. Patient returns to initial position and repeats. |  |  |
| **9. PUSH-UP** | 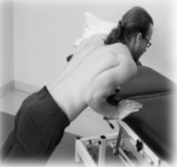 | |
| **EXERCISE:**  Initial position: Patient standing, shoulders at 90° of flexion with hands supported on a lower surface so the trunk is inclined at 45°. Execution: Push-up. Patient returns to initial position and repeats. |  |  |
| **10. RETRACTION OF THE SCAPULA – PRONE** | 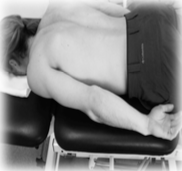 | |
| **EXERCISE:**  Initial position: Patient prone, upper limb by patient’s side. Neck in neutral position. Patient performs maximal elevation of shoulder and maximal depression of shoulder in order to find neutral position. Arm is then held there.  Execution: Retraction of scapula. Patient returns to initial position and repeats, without requiring stopping at mid-range each time. |  |  |
| **11. SHOULDER FLEXION WITH RESISTED EXTERNAL ROTATION** | 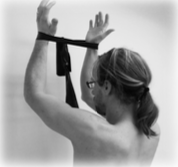 | |
| **EXERCISE:**  Initial position: Patient standing, shoulders at 30° of flexion, forearms neutral with an elastic band around the wrists.  Execution: Shoulder flexion through maximal pain-free range of motion. Patient returns to initial position and repeats. |  |  |
| **12. ASSISTED SHOULDER FLEXION** | 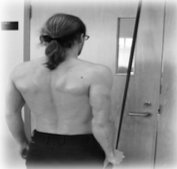 | |
| **EXERCISE:**  Initial position: Patient standing facing a door, trunk and legs parallel to the door. Patient pulls an elastic band hooked to the top of a door towards shoulder extension without exceeding neutral position of the shoulder (0°).  Execution: Let shoulders move towards flexion due to resistance of the elastic band. Patient returns to initial position and repeats. |  |  |
| **13. SCAPULA RETRACTION IN “U’’ POSITION – PRONE** | 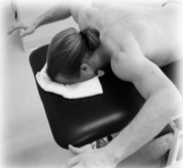 | |
| **EXERCISE:**  Initial position: Patient prone, arms at patient’s side. Neck in neutral position. Shoulders at 90° of abduction, forearms in pronation, and elbows at 90° of flexion. Hands are supported.  Execution: Retraction of scapula by performing horizontal abduction of the shoulders while lifting hands. Patient returns to initial position and repeats. |  |  |
| **Non-selected exercises for the SSE inventory** | | |
| **14. SHOULDER CIRCUMDUCTION – SIDE LYING** | 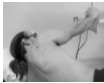 | |
| **EXERCISE:**  Initial position: Patient lying on their side, shoulder at 90° of flexion, elbow in extension, weight in hand. Protraction of the scapula.  Execution: Circumduction of the shoulder. |  |  |
| **15. SCAPULA MOBILIZATION – SIDE LYING** |  | |
| **EXERCISE:**  Initial position: Patient lying on their side, upper limb supported.  Execution: Protraction of the scapula. Patient returns to initial position and repeats. *  * *This exercise can be done in protraction, retraction, elevation and depression of the scapula.* |  |  |
| **16. PROTRACTION OF THE SCAPULA – SIDE LYING** | 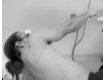 | |
| **EXERCISE:**  Initial position: Patient lying on their side, shoulder at 90° of flexion, elbow extended.  Execution: Protraction of the scapula. Patient returns to initial position and repeats. |  |  |
| **17. SHOULDER ELEVATION** | 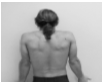 | |
| **EXERCISE:**  Initial position: Patient standing, shoulders at 30° of abduction.  Execution: Elevation of shoulders. Patient stops motion at mid-range between shoulder depression and full elevation. Patient returns to initial position and repeats. |  |  |
| **18. RESISTED RETRACTION** | 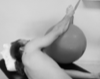 | |
| **EXERCISE:**  Initial position: Patient lying on their side, upper limb at 100° of flexion supported on a 21’’ Swiss ball, elastic band parallel to the upper limb, held in patient’s hand.  Execution: Retraction of the scapula. Patient returns to initial position and repeats. |  |  |
| **19. HORIZONTAL ABDUCTION OF THE SHOULDER** | 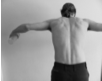 | |
| **EXERCISE:**  Initial position: Patient standing, hips slightly flexed with lumbar spine in neutral. Upper limbs perpendicular to the floor, elbows extended with weight in both hands. Execution: Horizontal abduction. Patient returns to initial position and repeats. |  |  |

| **Scapula motion definitions** | |
| --- | --- |
| **Upward rotation** | Glenoid cavity rotates in a cranial direction. |
| **Downward rotation** | Glenoid cavity rotates in a caudal direction. |
| **Anterior tilt** | Inferior angle of the scapula moves posteriorly while scapula rotates anteriorly. |
| **Posterior tilt** | Inferior angle of the scapula moves anteriorly while scapula rotates posteriorly. |
| **Depression** | Scapula glides inferiorly. |
| **Elevation** | Scapula glides superiorly. |
| **Protraction** | Medial border of scapula glides laterally. |
| **Retraction** | Medial border of scapula glides medially. |
